# Apelin Attenuates the Osteoblastic Differentiation of Vascular Smooth Muscle Cells

**DOI:** 10.1371/journal.pone.0017938

**Published:** 2011-03-18

**Authors:** Peng-Fei Shan, Ying Lu, Rong-Rong Cui, Yi Jiang, Ling-Qing Yuan, Er-Yuan Liao

**Affiliations:** 1 Department of Endocrinology and Metabolism, the Second Affiliated Hospital ZheJiang University College of Medicine, Hangzhou, Zhejiang, People's Republic of China; 2 Department of Endocrinology and Metabolism, People's Hospital of Zhongshan City, Zhongshan, Guangdong, People's Republic of China; 3 Institute of Metabolism and Endocrinology, The Second Xiang-Ya Hospital, Central South University, Changsha, Hunan, People's Republic of China; 4 Department of Pediatrics, The Second Xiang-Ya Hospital, Central South University, Changsha, Hunan, People's Republic of China; 5 Department of Pathology, The Second Xiang-Ya Hospital, Central South University, Changsha, Hunan, People's Republic of China; University of Queensland, Australia

## Abstract

Vascular calcification, which results from a process osteoblastic differentiation of vascular smooth muscle cells (VSMCs), is a major risk factor for cardiovascular morbidity and mortality. Apelin is a recently discovered peptide that is the endogenous ligand for the orphan G-protein-coupled receptor, APJ. Several studies have identified the protective effects of apelin on the cardiovascular system. However, the effects and mechanisms of apelin on the osteoblastic differentiation of VSMCs have not been elucidated. Using a culture of calcifying vascular smooth muscle cells (CVMSCs) as a model for the study of vascular calcification, the relationship between apelin and the osteoblastic differentiation of VSMCs and the signal pathway involved were investigated. Alkaline phosphatase (ALP) activity and osteocalcin secretion were examined in CVSMCs. The involved signal pathway was studied using the extracellular signal-regulated kinase (ERK) inhibitor, PD98059, the phosphatidylinositol 3-kinase (PI3-K) inhibitor, LY294002, and APJ siRNA. The results showed that apelin inhibited ALP activity, osteocalcin secretion, and the formation of mineralized nodules. APJ protein was detected in CVSMCs, and apelin activated ERK and AKT (a downstream effector of PI3-K). Suppression of APJ with siRNA abolished the apelin-induced activation of ERK and Akt. Furthermore, inhibition of APJ expression, and the activation of ERK or PI3-K, reversed the effects of apelin on ALP activity. These results showed that apelin inhibited the osteoblastic differentiation of CVSMCs through the APJ/ERK and APJ/PI3-K/AKT signaling pathway. Apelin appears to play a protective role against arterial calcification.

## Introduction

Arterial calcification, which is a common disease in diabetic and uremic patients, is associated with clinical complications, such as myocardial infarction, impaired vascular tone, angioplasty dissection, and poor surgical outcome [1], [2]. Recent data suggest that arterial calcifications are not a passive, degenerative, end-stage process of vascular disease, but the result of an active, regulated process. We and others have provided evidences that vascular calcification resembles osteogenesis [3]–[5], and factors regulating bone mineralization have been demonstrated in calcified plaques [6]. VSMCs contribute significantly to the active regulation of vascular calcification [7], [8]. However, the underlying mechanisms by which vascular calcification is regulated have not been fully understood as yet.

Apelin is a novel bioactive peptide identified as the endogenous ligand of the orphan G protein-coupled receptor, APJ [9], [10]. Apelin is synthesized as a 77 amino acid pre-pro-peptide that can be cleaved into fragments of different sizes that activate APJ. Apelin-13, which is active in forms that range in length from 13–36 residues, is the variant that is most active in signal transduction and consequently is most frequently studied. Apelin regulates body fluid homeostasis and cardiovascular functions through the apelin receptor, APJ, and the level of plasma apelin markedly increases in obesity that is associated with insulin resistance and hyperinsulinemia [11]–[14]. Recently, the apelin-APJ system has emerged as a potent regulator of cardiovascular function, mediating adaptations to physiological stress and disease. Apelin is involved in the propagation of action potentials and contractility in cardiomyocytes [15], as well as proliferation and myosin light chain phosphorylation in VSMCs [16]–[18]. Furthermore, our recent study demonstrated that apelin suppresses apoptosis of VSMCs [19]. These results show that the apelin-APJ system might play an important role in the vascular system. In a recent study, the apelin-APJ signaling system was showed to be involved in the regulation of aortic valve calcification [20], which is an actively regulated pathobiological process in a similar manner to vascular calcification. However, the effects of apelin on vascular calcification are still not known.

In the present study, the hypothesis was tested that the apelin-APJ signaling system is involved in the regulation of the osteoblastic differentiation of VSMCs. To elucidate the effect of apelin on the calcification of VSMCs and the mechanisms involved, we used calcifying vascular smooth muscle cells (CVMSCs), which are a specific subpopulation of vascular smooth muscle cells that can spontaneously express the osteoblastic phenotype gene and form calcification nodules [21]. We also examined the effect of apelin on the osteoblastic differentiation of CVSMCs and the cell signals pathway involved. This study provides new evidence that apelin directly modulates calcification of CVSMCs through the APJ/ERK and APJ/PI3-K/Akt signaling pathways, which suggests that targeting this peptide may provide a novel therapeutic avenue by which vascular calcification can be regulated.

## Results

### APJ was expressed in cultured CVSMCs

**Figure 1:**
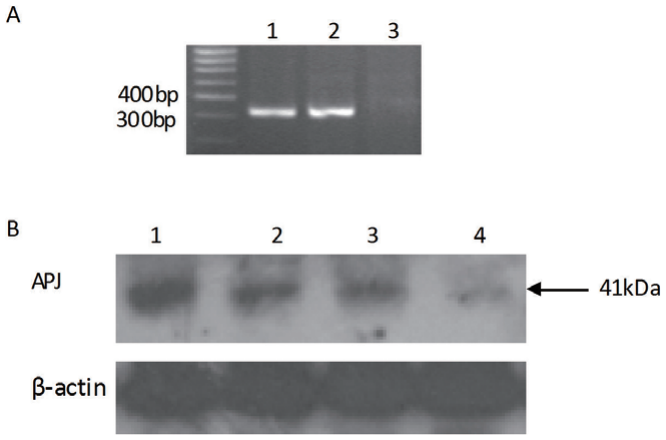
APJ expression in cultured CVSMCs. (A) APJ mRNA expression in CVSMCs. Total RNA from CVSMCs was subjected to RT-PCR. The PCR products were visualized in a 1.5% agarose gel stained with ethidium bromide. Lane 1 human subcutaneous adipose tissue as a positive control; Lane 2 CVSMCs; Lane 3 RNA of CVSMCs without RT as a negative control. (B) Representative results of Western blot analysis using an anti-APJ antibody. Total cellular protein was subjected to an immunoblotting analysis using anti-APJ antibody. The anti-APJ antibody identified a band at 41 kDa. Lane 1, human subcutaneous adipose tissue as the positive control; lane 2, lysate of CVSMCs; lane 3, lysate from CVSMCs treated with siRNA control; and lane 4, lysate from CVSMCs treated with siRNA-APJ. β-actin was used as the loading control.

Using RT-PCR, we confirmed that APJ mRNA was expressed in CVSMCs. APJ mRNA expression in human subcutaneous adipose tissue was used as a positive control (Fig. 1A). The results showed a 323 bp fragment specific to APJ. No bands were observed in reactions without RT as template. Using Western blot analyses, we confirmed that APJ proteins were expressed in CVSMCs; APJ expression in human subcutaneous adipose tissue was used as the positive control (Fig. 1). Treatment with siRNA-APJ significantly blocked the expression of APJ protein in CVSMCs since there were few bands detected, while no blockade was observed on treatment with the scrambled APJ siRNA. Our results demonstrated that CVSMCs primarily expressed APJ.

### Apelin attenuated the osteoblastic differentiation and mineralization of CVSMCs

**Figure 2:**
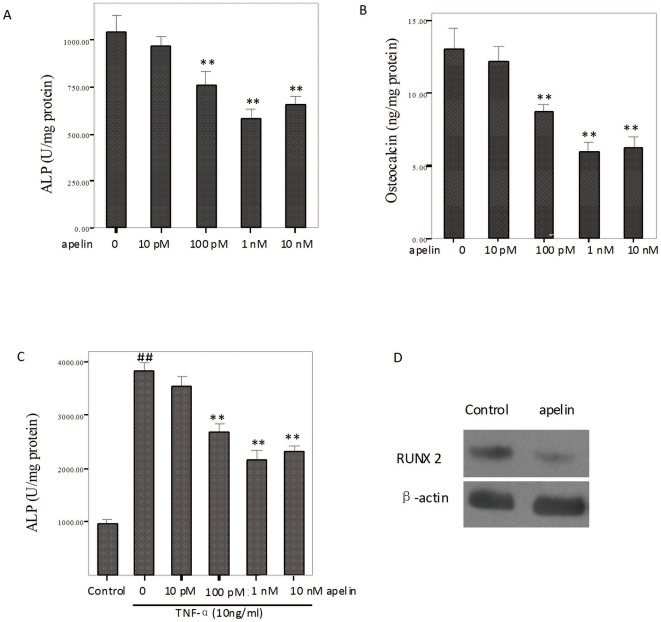
Effects of apelin on ALP activity, osteocalcin secretion and Runx2 protein expression in cultured CVSMCs. Cells were treated with vehicle (serum-free medium) or apelin at 10 pM, 100 pM, 1 nM, or 10 nM for 48 h in serum-free DMEM. ALP activity and osteocalcin secretion were determined and normalized to the total cell protein. (A) The dose response of apelin on ALP activity in cultured CVSMCs. (B) The dose response of apelin on osteocalcin secretion. The group results are shown. (C) The dose response of apelin on ALP activity with control (no TNF-a) or medium containing 10 ng/ml TNF-α in cultured CVSMCs. The bars represent mean ± SD (^##^ *P*<0.01 vs. control; **P<0.01 compared with CVSMCs given TNF-α induction without apelin, n = 3). (D) Cells were treated with vehicle (serum-free medium) or apelin at 1 nM for 48 h in serum-free DMEM. Total cellular protein was subjected to immunoblotting analysis using anti-RUNX2 and anti-β-actin antibody.

Recent data [3]–[5] suggested that arterial calcification resembles a bone mineralization process with expression bone-related molecules. Alkaline phosphatase and osteocalcin are well-established phenotypic markers of osteoblasts. We examined the activity of ALP and osteocalcin secretion during the osteoblastic differentiation of CVSMCs after stimulation by apelin. Treatment with apelin for 48 h significantly inhibited ALP activity in a dose-dependent manner. Figure 2 showed that the activity of ALP decreased significantly (P<0.01) at 10 pM, 100 pM, 1 nM, or 10 nM concentrations of apelin (Fig. 2A). Treatment with 100 pM, 1 nM, or 10 nM apelin caused a significant dose-dependent decrease in osteocalcin production (P<0.01; Fig. 2B). Treatment with 1 nM apelin caused a significant decrease in the expression of the Runx2 protein (Fig. 2D). Figure 2C shows that 10 ng/ml tumor necrosis factor-alpha (TNF-α) accelerated the activity of ALP, and the effects were attenuated by apelin in cultured CVSMCs (p<0.01; Fig. 2C).

**Figure 3:**
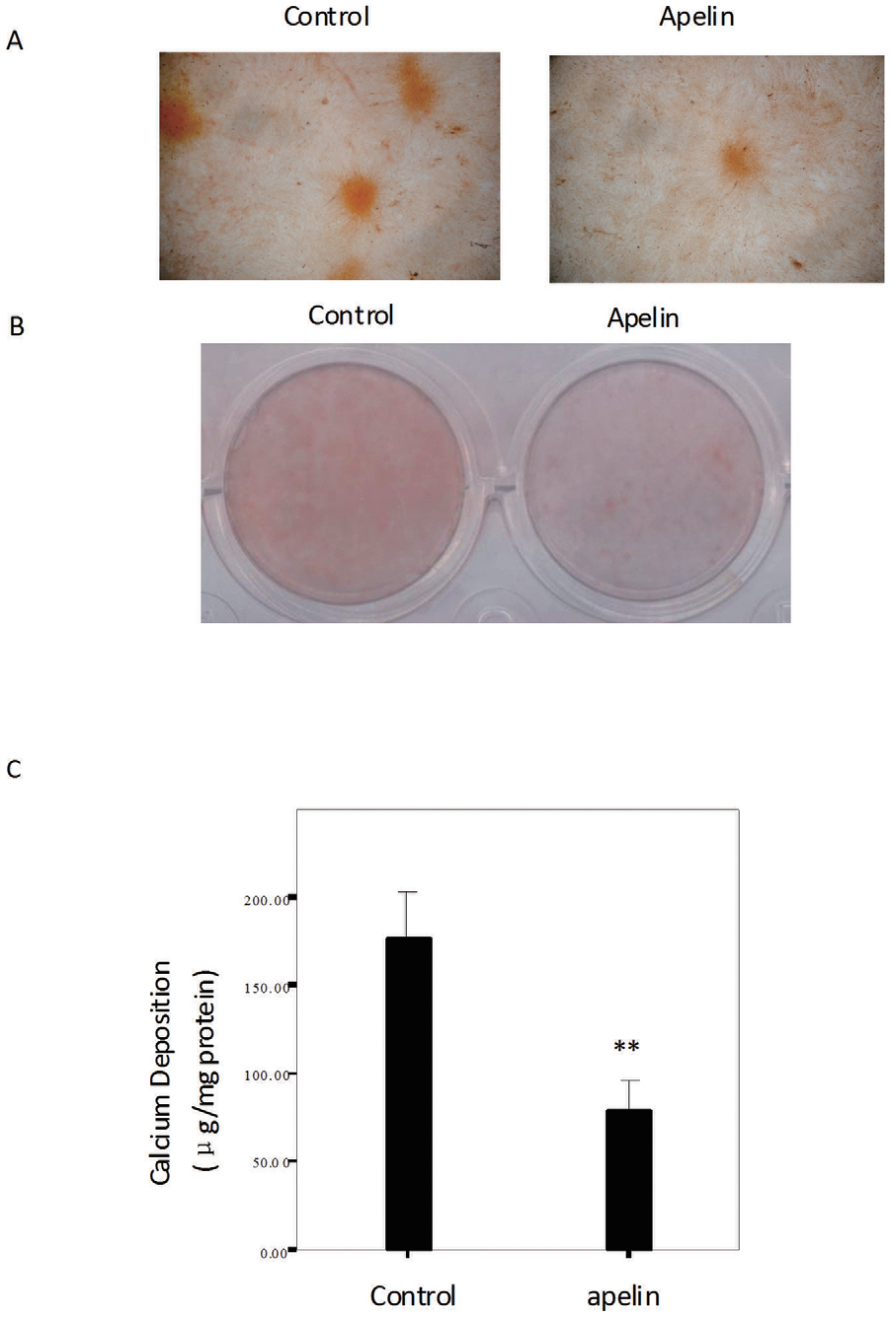
The effects of apelin on the mineralization of the matrix in cultured CVSMCs. (A) A representative microscopic view of the Alizarin Red S staining at a magnification of 400× for control cells and cells treated with 1 nM apelin in cultures of 12 d duration. (B) A representative entire plate view of the Alizarin Red S staining in 24-well plates for control cells and cells treated with apelin in 12 d cultures. (C) Quantification of the calcium content of the cell layers as measured by the atomic absorption spectroscopy method and normalized to the cellular protein content. The bars represent the mean ± SD (n = 3; **P<0.01 vs. control).

Matrix mineralization, a marker for osteoblastic differentiation and function, was assessed by the Alizarin Red S assay. Figure 3A shows a microscopic view of the Alizarin Red S staining, where apelin decreased mineralized nodule formation in 12 d cultures above that seen in control cultures. Figure 3B shows an entire plate view of the Alizarin Red S staining in 24-well plates, where treatment with apelin exhibited decreased Alizarin Red S staining after 12 days in culture compared to those that were not treated with apelin. Figure 3C shows that apelin decreased the calcification seen in CVSMCs, which was measured as CVSMCs calcium levels.

### Intracellular signaling mechanisms mediating apelin inhibition

**Figure 4:**
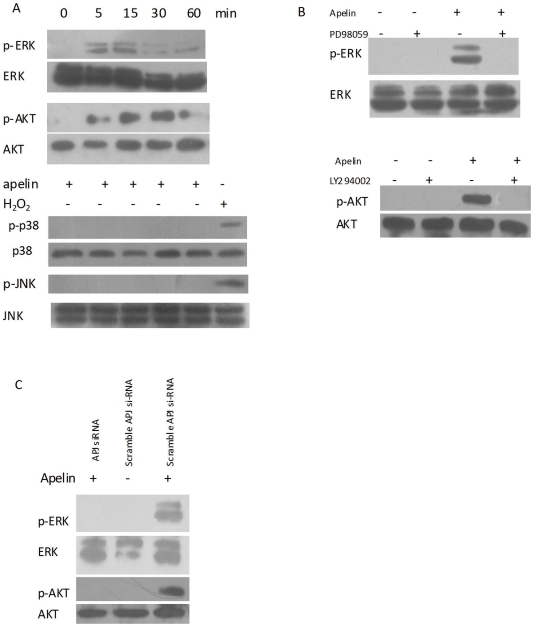
Effects of apelin on MAKP and PI3-K/Akt activation in CVSMCs. Cell lysates were subjected to Western blotting and incubated with antibodies against p-ERK, ERK, p-P38, P38, p-JNK, JNK, p-Akt, and Akt. The representative results are shown. (A) Cells exposed to 1 nM apelin for 5–60 min, or 50 µM H_2_O_2_ for 15 min as a positive control for P38 and JNK activation. (B) Cells incubated with PD98059 (10 µM) or LY294002 (10 µM) for 2 h prior to treatment with 1 nM apelin for 15 min. (C) Cells treated with scrambled APJ siRNA or APJ siRNA in the presence of 1 nM apelin.

Mitogen-activated protein kinase (MAPK) and PI3-K/Akt play an essential role in controlling cell differentiation. Therefore, we determined the level of MAPK and PI3-K/Akt signaling induced by apelin in CVSMCs. Apelin stimulated the activity of a specific MAPK, namely ERK, in CVSMCs 5 min after incubation began, as demonstrated by an increase in phosphorylated ERK (p-ERK) levels; the peak activation of ERK occurred at 15 min (Fig. 4A). Treatment with apelin also increased phosphorylated Akt (p-Akt) levels after 5 min of incubation (Fig. 4A). The peak activation of Akt occurred at 15 min. However, apelin had no effects on the activation of c-jun N-terminal kinases (JNK) or p38 MAP kinases (p38), and none of their phosphorylated forms were detected. To exclude the false negative results, we used 50 µM H_2_O_2_ as positive control, which could activate p38 and JNK in calcifying vascular cells [22].

Fig. 4B shows that the activation of ERK or Akt by apelin in CVSMCs was inhibited by the PD98059 (ERK inhibitor) or LY294002 (PI3-K inhibitor), respectively. Moreover, suppression of APJ with siRNA blocked the activation of ERK and Akt (Fig. 4C). These data indicate that apelin stimulates the ERK and PI3-K/Akt signal transduction pathways via APJ in CVSMCs.

### APJ/ERK and APJ/PI3-K/Akt signaling pathway mediated apelin decreased osteoblastic differentiation

In order to illuminate whether APJ, ERK, and PI3-K/Akt signaling was involved in the regulation of the osteoblastic differentiation of CVSMCs, we used siRNA to block the expression of APJ, and signal inhibitors to block the ERK and PI3-K/Akt signaling pathways, respectively. Then, we observed the activity of ALP, which was an important phenotypic marker of osteoblastic differentiation.

**Figure 5:**
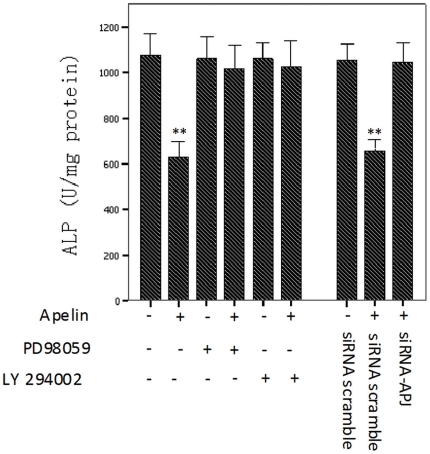
ERK and PI3-K signaling pathways mediated the apelin-induced osteoblastic differentiation of CVSMCs. Cell were incubated with PD98059 (10 µM) or LY 294002 (10 µM) for 2 h prior to treatment with 1 nM apelin. Cells were also treated with the siRNA control or siRNA-APJ in the presence of 1 nM apelin. ALP activity was measured, normalized to the total cellular protein contents. The bars represent the mean ± SD (n = 3; **P<0.01 vs. control).

Figure 5 showed that the pretreatment of cells with the ERK inhibitor, PD98059, and the PI3-K inhibitor, LY294002, blocked the increase in ALP activity that had been produced by 1 nM apelin. The APJ was efficiently knocked down by RNA interference in CVSMCs (Fig. 1). Suppression of APJ with siRNA-APJ, but not scrambled siRNA, also abolished the increased ALP activity by 1 nM apelin (Fig. 5). These data indicate that the apelin-reduced osteoblastic differentiation of CVSMCs is mediated by the APJ/ERK and APJ/PI3-K/Akt pathways.

## Discussion

The important findings of the present study were that apelin could directly attenuate the calcification of CVSMCs in vitro, which provided further evidence that vascular and bone calcification share regulatory factors. Apelin inhibited the mineralization of CVSMCs by decreasing the activity of ALP, an enzyme that has been shown to be important for matrix mineralization. Furthermore, apelin inhibited the osteoblastic differentiation of CVSMCs that is mediated by the APJ/ERK and APJ/PI3-K/Akt pathways. These findings demonstrate that CVSMCs can be direct targets of apelin.

Arterial calcification is an actively regulated process, which is similar to osteogenesis [23], [24]. The trans-differentiation process of VSMCs to the osteogenic phenotype plays a crucial role in the formation of arterial calcification. Calcifying vascular cells, a subpopulation of cells from the VSMCs that have the ability to spontaneously undergo osteoblastic differentiation and mineralization [5], is a very useful cell model for the analysis of the mechanisms of arterial calcification; this has been used in several studies [21], [25], [26]. Here, we employed CVSMCs to reveal the relationship between apelin and arterial calcification.

Apelin was found in 1998 to be an endogenous ligand of the human orphan G protein-coupled receptor, APJ, which previously had been identified by the Human Genome Project in 1993 as a member of the superfamily of 7-transmembrane G-protein coupled receptors [9], [10]. Apelin peptides have been shown to affect many biological functions in mammals, including the neuroendocrine, cardiovascular, and immune systems [27]. Apelin and APJ are widely expressed throughout the body [28]–[31]. In vascular system, apelin and APJ are known to be expressed in the endothelium and VSMCs [32], [33], which indicates a paracrine or autocrine mode of action in the vascular system. Recently, Hashimoto et al. reported that apelin induces the phosphorylation of the myosin light chain, which is a major regulatory event in initiating smooth muscle contraction [18]; apelin was also found to stimulate the proliferation of VSMCs, which indicates a possible function role for apelin in vascular function. Some experiments have shown that apelin elicits endothelium-dependent, nitric oxide-mediated vasorelaxation and reduces arterial blood pressure [34], [35], as well as endothelium-independent vasoconstriction, by acting directly on VSMCs [36]. In addition, apelin demonstrates potent and long-lasting positive inotropic activity, which is preserved even in injured myocardium and is not accompanied by myocardial hypertrophy [37], [38]. These results indicate that apelin has a protective effect on the cardiovascular system.

Our previous study demonstrated that apelin inhibited the apoptosis of VSMCs through the APJ/PI3-K/Akt pathway [19]. However, the effect of apelin on osteoblastic differentiation by VSMCs is still unknown. Using RT-PCR and immunoblotting, we identified CVSMCs that primarily expressed APJ and were a target for the action of apelin. Our data showed that apelin decreased the activity of ALP and osteocalcin secretion, which are well-recognized as early markers of osteoblastic differentiation and increase during the differentiation of CVSMCs. Meanwhile, apelin directly inhibited the formation of mineralized nodules. We also showed that TNF-α increased the activity of ALP, and this effect was attenuated by apelin treatment in cultured CVSMCs. These results appear to contradict our previous study, which showed that apelin had no effect on the activity of ALP in human osteoblasts [39]. We speculate that this might be a result of the use of different kinds of cells used in each study. Our present study also demonstrated apelin decreased the expression of Runx2 in CVSMCs, which is an important osteoblast differentiation transcription factor. These results suggest apelin inhibit osteoblastic differentiation of CVSMCs.

Several cytokines [40]–[46] have been identified as playing biphasic effects on the osteoblastic differentiation of VSMCs and osteoblast differentiation. For example, adiponectin could increase osteoblast differentiation [41], [47] and decrease the osteoblastic differentiation of VSMCs [21]. Furthermore, vascular calcification is frequently accompanied by decreased bone mineral density or disturbed bone disease. This contradictory association, which has been observed mainly in osteoporosis and chronic kidney disease, is called the ‘calcification paradox’ [48]. Here, our present data demonstrated that apelin directly inhibited the osteoblastic differentiation of CVSMCs.

To gain further insight into the mechanism by which apelin inhibited the osteoblastic differentiation of CVSMCs, we evaluated signaling pathway events. MAPK and PI3-K/Akt are well known to play essential roles in controlling cell differentiation [49]–[51]. Our study showed that apelin induced the activation of ERK and Akt in CVSMCs. Treatment with the PI3-K inhibitor, LY294002, abolished the activation of Akt in human VSMCs, which indicated that the phosphorylation of Akt is dependent on PI3-K. The suppression of APJ with siRNA blocked the effects of apelin on ERK and Akt, which suggested that the activation of ERK and PI3-K/Akt was mediated through APJ. Furthermore, the inhibition of APJ expression, PI3-K/Akt activation, or ERK activation, blocked effects of apelin on decreasing the activity of ALP. This indicates that apelin inhibits the osteoblastic differentiation of CVSMCs through the APJ/ERK and APJ/PI3-K/Akt signaling pathways.

Other studies as well as our previous experiments demonstrated that apelin activates the ERK and PI3-K/Akt pathways [19], [52]. These stimulations are generally recognized as characteristic of cardioprotective agents. Several studies [6], [53], [54] have indicated that PI3-K/Akt activation inhibited the osteoblastic differentiation of VSMCs, and the results of our present experiment supported this conclusion. The relationship between ERK activation and osteoblastic of VSMCs is still controversial. Several studies have shown that ERK activation might obscure the osteoblastic differentiation of VSMCs [4], [55], and others results have been inconsistent with these [55]–[57]. Meanwhile, other experiments have concluded that ERK activation had no effect on the osteoblastic differentiation of VSMCs [58]. Our present study also showed that the ERK inhibitor, PD98059, attenuated the activity of ALP that had been decreased by apelin. It is necessary that the relationship between ERK phosphorylation and osteoblastic differentiation of VSMCs should be thoroughly investigated.

Our findings suggest a crucial role for apelin in decreasing the osteoblastic differentiation of VSMCs, and the APJ/ERK and APJ/PI3-K/Akt is implicated as being involved in this effect. This suggests that apelin plays a protective role against arterial calcification.

## Materials and Methods

### Reagents

Synthetic apelin-13 peptide was purchased from the American Peptide Company Inc. (Sunnyvale, CA, USA). The amino acid sequence of apelin-13 is Gln-Arg-Pro-Arg-Leu-Ser-His-Lys-Gly-Pro-Met-Pro-Phe. Recombinant human IGF-I protein was purchased from PEPROTECH (London, UK). Anti-human RUNX2 (also called cbfa1, PEBP2aA) antibody, anti-extracellular signal-regulated kinase (ERK), p-ERK, p-p38, p38, c-jun N-terminal kinase (JNK), p-JNK, Akt, p-Akt antibody, anti-mouse, and rabbit IgG peroxidase conjugate antibodies were purchased from Santa Cruz Biotechnology Inc (Waltham, MA, USA). Anti-human apelin receptor (APJ) antibody was purchased from Abcam Inc. (Cambridge, MA, USA). Anti-β-actin polyclonal antibody was purchased from Sigma (St. Louis, MO, USA). PD98059 and LY294002 were purchased from Calbiochem Corp. (San Diego, CA, USA).

### Cell culture and in vitro calcification

Human calcifying vascular smooth muscle cells (CVSMCs) from excess donor vasculature after kidney transplantations were grown from explants of normal arterial tissues, and approved by the Ethics Committee of the Second Xiangya Hospital of Central South University, China. Written informed consent was obtained from each participant. The cells were cultivated as we have previously described [21], and experiments were performed between passages 3 and 6 from the primary culture. Briefly, the arterial tissues were removed under sterile conditions. After the arterial tissues were rinsed several times in Hanks's balanced salt solution, the adventitia was removed; the remaining arterial tissues were minced and digested in 5 ml of digestion solution (0.125 mg/ml elastase, 0.25 mg/ml soybean trypsin inhibitor, 10 mg/ml collagenase I, 2.0 mg/ml crystallized bovine albumin, and 15 mM HEPES) at 37°C for 45 min. The cellular digests were filtered through a sterile 100-mM nylon mesh, centrifuged at 1,000 rpm for 10 min, and washed twice in Dulbecco's modified Eagle's medium (DMEM) containing 10% FBS (Gibco-BRL) before culture in the same medium. CVSMCs were isolated from cultures in which multicellular nodules spontaneously appeared. From these nodule-forming cultures, cells were cloned by limiting dilution and single cell harvesting. Colonial lines were identified as CVSMCs by their positive staining with monoclonal antibody α-actin and by their ability to express high levels of ALP and form calcified nodules.

### Adipose tissue

Subcutaneous adipose tissue was obtained from patients who had undergone mammoplastic surgery. Samples were snap-frozen and stored in liquid nitrogen until the extraction of proteins.

### Detection of APJ gene expression in cultured CVSMCs by RT-PCR

To investigate the expression of APJ mRNA in CVSMCs, reverse transcription polymerase chain reaction (RT-PCR) was performed as our previous described [39]. Total RNA from cultured CVSMCs and subcutaneous adipose tissue was isolated using Trizol reagent (Gibco) according to the manufacturer's recommended protocol. There is only 1 exon and 1 gene found in the APJ genomic region spanning 4535 bp. We could not obtain intron-spanning primers for APJ, therefore we performed DNase I digestion of isolated total RNA from cultured CVSMCs and subcutaneous adipose, to ensure no genomic contamination. The PCR primers were 5′cacctggtgaagacgctgta 3′ and 5′ taggggatggatttctcgtg yielding a 323 bp fragment. CVSMCs RNA without RT as negative control was included. RT was performed using 1.0 µg total RNA and the Reverse Transcription System (Promega, Madison, WI, USA). PCR was performed as follows: 94°C for 1 min, 58°C for 45 s, and 72°C for 1 min for 35cycles followed by a 10 min incubation at 72°C. Ten microliters of the reaction mixture was electrophoresed on 1.5% agarose gels and stained with ethidium bromide to verify bands. The identities of PCR products were confirmed by direct sequencing using an automatic DNA sequencer (PE Applied Biosystems).

### Detection of APJ in cultured CVSMCs by immunoblotting analyses

To investigate the expression of APJ protein in CVSMCs, the cell layers were homogenized with Triton lysis buffer (50 mM Tris-HCl, pH 8.0, containing 150 mM NaCl, 1% Triton X-100, 0.02% sodium azide, 10 mM EDTA, 10 mg/ml aprotinin, and 1 mg/ml aminoethylbenzenesulfonyl fluoride). Homogenized protein (50 mg) was loaded onto a 7.5% polyacrylamide gel. After electrophoresis, the SDS-PAGE-separated proteins were transferred onto a nitrocellulose membrane (Amersham Pharmacia Biotech). The membrane was blocked with 2.5% nonfat milk in PBS and incubated with anti-mouse APJ in PBS for 2 h. The membrane was reprobed with rabbit anti-mouse IgG conjugated with horseradish peroxidase for 1 h. Blots were processed using an ECL kit (Santa Cruz) and exposed to the film. The same membrane was stripped and reprobed with anti-β-actin antibody as a loading control.

### Analysis of alkaline phosphatase activity and osteocalcin secretion in cultured CVSMCs

Calcifying CVSMCs were washed three times with PBS, scraped into 1 ml of 10 mM Tris-Cl buffer (pH 7.6) that contained 0.1% Triton-X-100 on ice, and centrifuged. The lysates were homogenized. Then, ALP activity was assayed by the spectrophotometric measurement of p-nitrophenol release at 37°C. ALP activity was normalized to the total protein content of the cell layer. Osteocalcin released into the culture media was measured using a specific radioimmunoassay kit (DiaSorin, Stillwater, MN, USA). To normalize protein expression to the total cellular protein, a fraction of the lysate solution was used in a Bradford protein assay.

### Measurement of mineralized matrix formation

For Alizarin Red S staining, CVSMCs in 24-well plates were cultured in medium that contained either 1 nM apelin or vehicle for 12 days. Then, the extent of mineralized matrix was determined by Alizarin Red S staining [59]. Briefly, cells were fixed in 70% ethanol for 1 h at room temperature and stained with 40 mM Alizarin Red S for 10 min. Next, cell preparations were washed with PBS to eliminate nonspecific staining. The stained matrix was assessed using a Nikon Diaphot inverted microscope and samples were photographed using a Nikon 35-mm camera (Nikon, Tokyo, Japan).

For the quantification of calcium levels, cells were washed with PBS and decalcified with 0.6 N HCl for 24 h, Calcium content was determined by measuring the concentrations of calcium in the HCl supernatant by atomic absorption spectroscopy. After decalcification, the cells were washed three times with PBS and the cells were solubilized with 0.1 N NaOH/0.1% SDS. The protein content was measured with a Bradford protein assay. The calcium content of the cell layer was normalized to the protein content.

### RNA interference against APJ

Two pairs of small-interfering RNAs (siRNAs) and control siRNAs were synthesized by Genesil Biotechnology Co. (Wuhan, China) as previously described [39]. The targets were GGUGCAGUGCUACAUGGACdTdT (for human APJ siRNA) and AUGCUGCGAGCUAGAUCGGdTdT (for scrambled human APJ siRNA). For gene knockdown experiments, CVSMCs were plated into 60-mm-diameter dishes and cultured for 24 h in medium without antibiotics. Cells were transfected with siRNAs (1 nmol per well) using Lipofectamine 2000 (Invitrogen Inc. Carlsbad, CA, USA) according to the instructions of the manufacturer. After 24 h of culture, cells were retransfected with siRNAs or controls and then recultured for another 48 h. Protein expression was analyzed by Western blotting.

### Detection of MAPK and PI3-K/Akt activation by Western blot analysis

CVSMCs were initially treated with 1 nM apelin for between 5–60 min. Then, cellular monolayers were washed quickly with cold PBS that contained 5 mM of EDTA and 0.1 mM Na_3_VO_4_, and lysed with a lysis buffer that consisted of 20 mM Tris–HCl (pH 7.5), 150 mM NaCl, 1% Triton X-100, 10 mM of NaH_2_PO_4_, 10% glycerol, 2 mM Na_3_VO_4_, 10 mM NaF, 1 mM ABSF, 10 mg/ml leupeptin and 10 mg/ml aprotinin. Western blotting was performed as described above. Proteins were then transferred to a nitrocellulose membrane. The membrane was incubated with anti-p-p38, -p38, -p-ERK, -ERK, -p-JNK, -JNK, -p-Akt and -Akt primary antibodies at 1∶500 in PBS for 2 h. The membranes were then incubated with goat anti-mouse or rabbit IgG antibody conjugated with horseradish peroxidase at 1∶1000 in PBS for 1 h. Blots were processed using an ECL Kit (Santa Cruz) and exposed to X-ray film.

### Detection of signaling pathways involved in the apelin-attenuated osteoblastic differentiation of CVSMCs

To determine the downstream intracellular signaling pathways affected by apelin treatment, we explored the relationship between extracellular signal-regulated kinases (ERK) and PI3-K phosphorylation of CVSMCs and treatment with apelin. CVSMCs were pretreated with 10 µM PD98059 (an ERK inhibitor) or 10 µM LY294002 (a PI3-K inhibitor) for 2 h prior to apelin treatment. ALP activities were assessed after 48 h.

### Statistical analyses

The results of the experiments were normalized relative to total protein levels, as determined by Bradford's method. The data are expressed as means ± standard deviation (SD). Comparisons between values of more than two groups were performed by an analysis of variance (one-way ANOVA). P-values of less than 0.05 were considered to be statistically significant in all cases. All experiments were repeated at least three times, and representative experiments are shown in the Figures.
